# Ecological and phylogenetic aspects of the spring diet of three palaearctic species of swans

**DOI:** 10.1186/s12862-024-02204-7

**Published:** 2024-02-02

**Authors:** Sergei A. Kouzov, Anna V. Kravchuk, Elena M. Koptseva, Yulia I. Gubelit, Elmira M. Zaynagutdinova, Evgeny V. Abakumov

**Affiliations:** 1 Department of Applied Ecology, St. Petersburg State University, Universitetskaya Emb. 7-9, St. Petersburg, 199034 Russia; 2 Department of Vertebrate Zoology, St. Petersburg State University, Universitetskaya Emb. 7-9, St. Petersburg, 199034 Russia; 3 Zoological Institute RAS, Universitetskaya Emb. 1, St. Petersburg, 199034 Russia

**Keywords:** Feeding ecology, Diet, Fecal analysis, Mute swan, Bewick’s swan, Whooper swan

## Abstract

The quality of swans' nutrition at spring migration stopovers is important for their successful breeding. It is of great interest to study the differences in nutrition of different swan species when sharing the same habitat. Microscopic analysis of *Cygnus olor, C. cygnus*, and *C. columbianus bewickii* feces collected in the eastern part of the Gulf of Finland in February-April 2014–2019 was performed. We measured food preferences of the three swan species using non-metric multidimensional scaling (NMDS). The width and overlap of dietary niches were also calculated. The diet of *C. olor* consists almost entirely of soft submerged aquatic vegetation, mainly macroalgae. Samples of the other two species except macroalgae contained large amounts of young shoots and roots of rigid semi-submerged and coastal vegetation. The dietary niche of *C. cygnus* is the most isolated because it is dominated by thick rhizomes of *Phragmites australis*, which are hardly used by other swan species. The diet of Bewick’s swans was similar in many respects to that of the Mute swan, but Bewick’s swans much more often preferred vegetative parts of submerged and semi-submerged plants, such as *Stuckenia pectinata, Potamogeton perfoliatus, Sparganium sp., Nuphar lutea*, and others. Notably, the dietary niches of Mute swan and Whooper swan overlapped as much as possible in February March during a period of severe food shortage, in contrast to later periods in spring when food was more abundant and varied. In general, differences in diets are well explained by differences in the morphology of birds. Comparison of tarsometatarsus indices shows that *C. olor* is the most water-related species. *C. olor* has the longest neck and its beak has the strongest filter features, whereas beaks of the other two species shows noticeable “goose-like grazing” features. Moreover, *C. Cygnus* has the most powerful beak. These features are due to the history of species. The formation of *C. olor* occurred during the Miocene-Pliocene of the Palaearctic in the warm eutrophic marine lagoons of the Paratethys with abundant soft submerged vegetation. The evolution of *C. cygnus* and *C. c. bewickii* took place in Pleistocene. At that time, periglacial and thermokarst water bodies on permafrost became widespread in the Palearctic, as well as dystrophic peat lakes with much poorer submerged aquatic vegetation, but well-developed coastal and semi-submerged vegetation.

## Background

Swan diets have been studied by researchers for a very long period of time [1–6]. The greatest number of publications on this subject is devoted to two species: the Mute swan *Cygnus olor* [7–13] and the Bewick’s swan *Cygnus columbianus bewickii* [10, 14–17]. However, the vast majority of data on Mute swan feeding were obtained at various inland water bodies of Western Europe (see above), and the data on Bewick’s swan feeding mainly reflects the situation on wintering grounds [10, 14–16, 18, 19].

Unlike the Whooper swan *Cygnus cygnus* and the Bewick’s swan, the Mute swan has almost no rigid terrestrial or coastal vegetation in its diet (families Cyperáceae, Poáceae, Juncaceae) [2–4, 20], on the other hand, multicellular green, red, and brown algae are well represented among the submerged vegetation they consume [3]. The Mute swan switches to feeding on animal food in exceptional cases when traditional food is not available at wintering grounds. In the northern Black Sea region, cases of feeding on fish are known [21]. During winter on the Polish coast of the Baltic Sea, Mute swans feeding on mollusks *Dreissena polymorpha* have been revealed [22].

In the diet of the other two species (Whooper swan and Bewick's swan), in addition to a vascular submerged vegetation, semi-submerged and coastal plants was revealed. Small aquatic invertebrates were also present [1–6, 20, 23, 24]. Wintering in Western Europe, these species feed mainly on *Zostera marina*, *Ruppia maritima, Zannichellia palustris* and *Chara sp*. at the seaside [2–4, 6], and *Potamogeton pectinatus* tubers in inland water bodies [2–4, 10, 14–16, 18, 19, 25]. In recent decades, these two species have become wintering on root crops (mainly potatoes) on farmland [14–18, 26–28], as well as on corn (maize), winter rapeseed, and barley fields [29, 30].

In Poland, all three swan species show a marked preference for cornfields [31]. The utilization of new forage resources on wintering grounds may be partly related to the growth of the Whooper and the Mute Swan populations and increased competition in the traditional wintering grounds of swans. Thus, carbohydrate-rich feeds such as *Potamogeton pectinatus* tubers and *Chara sp.* nodules, as well as agricultural root crops and grains play an important role in the nutrition of these species during wintering.

During spring migrations in the Baltic Sea, we only know the study of Mute swans' faecal samples and stomachs obtained in the 1950s (Baltic coast of southern Sweden (Blekinge)), 25 plant species were found here [32]. The diet consisted mainly of *Ruppia sp*., *Potamogeton pectinatus*, *Pylaiella littoralis*. Somewhat less frequently, birds ate *Myriophyllum spicatum, Zostera marina, Zannichellia palustris, Najas marina, Ranunculus baudotti, Chara sp., Cladophora sp., Enteromorpha sp*. (ibid.).

**Fig. 1:**
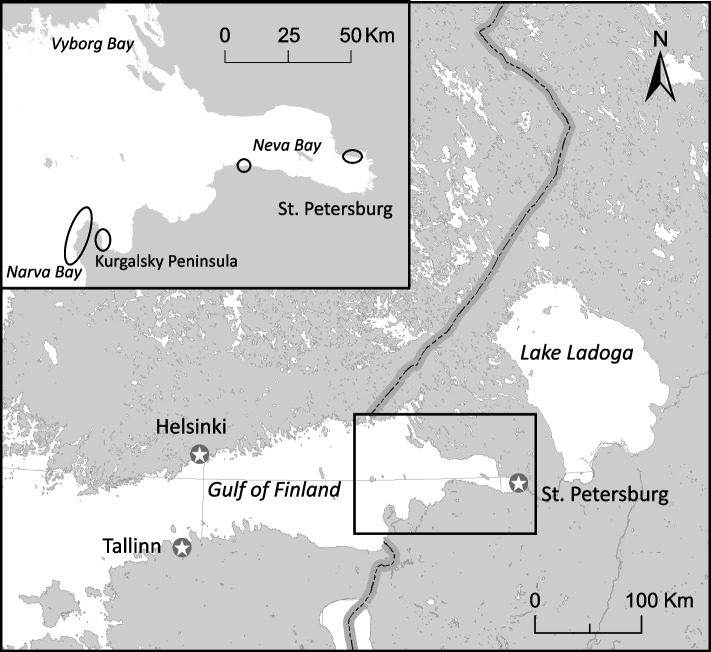
Study area and sites where fecal samples were collected (marked with circles). Data Source—SRTM: NASA, NGA, ESRI; GTOPO30: DCW, USGS EROS, ESRI

However, such studies have never been carried out in the eastern sector of the Baltic Sea, including the eastern part of the Gulf of Finland. Ecological conditions here differ significantly both from those of typical marine sites in the western Baltic and from those of freshwater water bodies. Here is a zone of transition from the freshwater river estuary of Neva Bay to the brackish open water area of the central part of the Gulf of Finland (Fig. 1). Therefore, the species composition of aquatic vegetation is impoverished - many species of both typically freshwater and typically marine vegetation are absent. Nevertheless, this area plays an important role in migrations of waterbirds [33]. Here are the largest migratory stopover sites of swans, where birds accumulate energy reserves for long migration to the next stop in the Northern Dvina delta and then - to the breeding grounds [33–35].

It is worth mentioning, that herbivorous Anseriformes change their feeding preferences in spring from the carbohydrate diet typical for autumn migrations and wintering, they switch to feeds rich in proteins necessary for clutch formation. For example, arctic geese (*Branta leucopsis, Anser albifrons and Anser fabalis)* follow the "green wave" of young sprouts of protein-rich cereals in spring [36–39]. We assume similar seasonal changes in swans. Therefore, it is interesting to find out what food resources are used by swans at spring migratory stopovers?

Mute swan, Whooper swan and Bewick’s swan have substantial differences in ecology, morphology, distribution, and history of speciation [2, 3, 6]. However, we are aware of only one study on the differences in swan’s diets when sharing a habitat [40]. Unfortunately, almost all other published data are derived from autecological studies conducted in different places and habitats. Therefore, it is not entirely clear whether these dietary differences are related to the presence or absence of certain foods in the studied areas or to the stable preferences of species associated with their ecology, morphology and evolutional history?

The aim of this work was to determine the main feeding resources, differences in diets and the degree of divergence in dietary niches among the three Palearctic swan species at their joint spring migration sites in the eastern part of the Gulf of Finland. We also attempted to explain the differences in food preferences of the species based on the features of their morphology, history of speciation and paleoecological data.

### Phylogeny and paleontological history of the studied species

The Mute swan (*Cygnus olor Gmelin* 1789) occupies a separate place from other Holarctic swans in the taxonomy of the genus *Cygnus* [41–45].

The Mute swan together with the Black swan *C. atratus* and the Black-necked swan *C. melanocoryphus* is included in the subgenus *Cygnus* [41]. These are the most ancient, well morphologically differentiated species, the evolution of which is connected with the tropical and subtropical zone of the Old and New Worlds [41, 46]. The last two species are classified by some taxonomists as separate subgenera *of Chenopsis* and *Sthenelides* [47], which is an additional argument for the antiquity of this group [3, 46, 47].

Fossil forms related to subgenus Cygnus, for example *C. mariae* and *C. paloregonus* are known from finds in North America (California, Idaho, Oregon, Arizona) from the Miocene to the Late Pleistocene [48–51]. In the Holocene, there is already no evidence of the presence of swans of the subgenus *Cygnus* in North America.

*Cygnus pristinus* is very close to the Mute swan, it is known in the Palearctic from the Late Miocene and Early Pliocene of Mongolia [52]. The Mute swan was found in the Miocene of the Northern Black Sea region [53], and the Late Pleistocene of Azerbaijan [54]. In the Late Pleistocene and Holocene, finds of Mute swan fossils were noted in many areas of Western and Eastern Europe: on the south of European Russia [53], in France [47], in East Anglia [55], Ireland, Portugal and Italy [56].

The Whooper swan and the Bewick’s swan together with the Nearctic forms the Trumpeter swan *C. biccinator* and the American tundra swan *C. columbianus* constitute the subgenus Olor. Different taxonomists interpret these forms differently [17, 57] from separating them into four separate species [6, 58–61] to combining these forms (as subspecies) into one [62], two [63–65] or three species [66, 67]. This indicates a fairly young age of these species [46, 57].

*Cygnus verae* from the early Pliocene of Bulgaria appears as transitional form between the subgenus *Cygnus* and *Olor* [68]. Probably, the main evolution of swans of the subgenus *Olor* took place in the Pleistocene, because fossil remains of species of this group are known only from this period [46, 57, 69]. *C. equitum* and *C. falconeri* are known from Malta and Sicily in the Middle Pleistocene [70, 71]. Fossils of *C. cygnus* are common from the Pleistocene and Holocene of the Black Sea, southern Ukraine, European Russia [53], and southern Siberia [72]. Fossils of *C. c. bewickii* are known from the Pleistocene and Holocene of southern Siberia [72]. In North America*, C. hibbardi*, similar to *C. columbianus* existed in early Pleistocene [73]. Fossils of *C. columbianus* are known from Late Pleistocene and Holocene [74–78], *C. bicinator* from Pleistocene [75, 79, 80].

### Main ecologically significant features of the swans’ morphology

The Mute Swan has the highest body mass among Palaearctic swans [2, 4] and the lowest wing length index and tarso-metatarsus length index relative to body mass. [2, 4, 81]. These indices suggests that the Mute swan among other Palearctic swans is the form most associated with water and the closest migrant [2, 81]. The latter is also associated with the smallest pneumatization of wing bones in this species [81]. The Whooper Swan and Tundra Swan are more terrestrial species (compared to the Mute Swan), better adapted to long-distance flights. The latter is especially pronounced in the most diminutive species, the Tundra Swan, which has the highest index of wing length [81].

In the beak morphology of the Mute swan, filtrating features are most pronounced [82]:

1. The largest number of filter lamellae (among this group of species) is on the jaws, especially on the upper row of the mandible, where their number is almost twice as large as in other Holarctic species [83].
2. The smallest absolute and relative sizes of the maxilla and mandible nails [83].
3. The highest density of mechanoreceptors at the end sections of the beak and the largest sizes of tactile bodies and holes [83].

In the morphology of the beaks of the Bewick’s swan and the Whooper swan, on the contrary, “grazing features” are noticeably expressed, bringing them closer to the structure of the beaks of geese [82, 83], the main differences between these two species are only in the linear dimensions of the beaks, which determines the power of the beak (ibid.).

### Distribution, subspecies, populations and population trends

The Mute Swan is a monotypic species, and its natural range covers a strip of semi-deserts, southern and typical steppes of Eurasia from the Black Sea to the Far East, as well as the forest zone of Western and Central Europe [2, 6, 57, 84–86]. In Eurasia, it forms several geographic populations: the British Isles population, the Western and Central European population, the Black Sea and Eastern European population, the Caspian Sea, Western Siberia and Central Asia populations, and the Eastern Asian population [2, 57, 87]. Swans from the Western European population were present in our study area. This population has shown exponential population growth and range expansion to the northeast in recent decades [88–91].

The Whooper Swan is also a monotypic species that inhabits forest-steppe, nemoral and boreal forests and forest-tundra from Scandinavia to the Far East [1–6, 92]. It forms several large geographic populations [57]: the Icelandic and British Isles population, the Scandinavian and North-Eastern European population, the Ural and Western Siberian population, the Central Siberian population and the East Asian population. Birds of the Scandinavian and North-Eastern European populations were observed in our study area. This population has experienced strong population growth and southward range expansion in recent decades [31, 57, 93–97].

The Bewick's Swan is currently distributed in forest tundra, southern and typical tundra from the Kanin Peninsula to Chukotka [2, 25, 60, 98, 99]. It is now considered by most taxonomists to be one of two subspecies of the Tundra Swan *C. columbianus* [63, 98, 100–102]. At the same time, the presence of a number of morphological differences between *C. c. columbianus* and *C. c. bewickii* [6, 43], and, most importantly, the almost complete absence of hybridization between them in the sympatric area of Chukotka [25, 59] support the separation of these forms into distinct species [25, 59]. The Bewick's Swan *C. c. bewickii* forms two large geographic populations—western and eastern [25, 59]. Previously, these populations were treated as subspecies of *C. bewickii bewickii* and *C. bewickii jankowskii* [2, 6, 25, 60]. The zone of intergradation of these subspecies in the twentieth century was considered to be the area between the Taimyr Peninsula and the Lena River delta [6, 60]. Currently, the western population of *C. c. bewickii* is significantly declining in numbers and distribution area, while the eastern population is expanding its range westward [25, 103]. The exact boundaries separating their distribution areas are still not clear enough. The migration routes of the western population pass through our study area [25]. Occurrence of individuals of the eastern population at wintering and migration stopover sites in Western Europe and the Baltic Sea is considered possible but unproven [59]. The observations of "black-billed" individuals cannot be considered as precise evidence due to the significant polymorphism of beak coloration in this species [59]. The European population of the Tundra Swan has declined by approximately 24% over the last 35 years and its conservation status is currently categorized as 'Vulnerable' (VU) by the IUCN [103].

## Methods

### Study area

In the eastern part of the Gulf of Finland (Fig. 1), there is a gradual transition from the freshwater shallow estuary of the Neva River with a dominance of a sandy hydroaccumulative landscape in the east to the deep brackish-water marine area in the west [104–106]. Another important feature of the region is the transition from a moraine landscape in the south to a selga rocky landscape in the north [104, 107, 108]. Compared to other parts of the Baltic Sea, the eastern part of the Gulf of Finland has a low salinity, which varies from 0.2‰ in the Neva Bay to 7.1‰ off the Kurgalsky Peninsula and the islands of the central part of the Gulf of Finland [109].

The coastal zone of the Neva Bay is characterized by extensive marshes with a rich species composition of vascular submerged and semi-submerged vegetation, similar to freshwater eutrophic water bodies. To the west of the Neva Bay, most of the purely freshwater vascular plants disappear due to increased salinity. Thickets of *Phragmites australis, Schoenoplectus tabernaemontani,* and *Bolboschoenus maritimus* are abundant in places sheltered from storms. Among freshwater submerged vegetation, *Zannichellia palustris, Stuckenia pectinata* and *Potamogeton perfoliatus* are mainly present. Separate clumps of marine vascular vegetation appear—*Ranunculus marinus*, *Ruppia maritima* and *Nájas marina* [110].

Salinity is one of the main factors determining the composition and distribution of macroalgae [111]. Therefore, the algal flora of the Russian part of the Gulf of Finland is an extremely depleted version of the Baltic macroalgae flora: 22 species of Chlorophyta, 15 species of Phaeophyta and 7 species of Rhodophyta [112, 113]. During the summer, macroalgae form accumulations. Some of them remain in coastal area, and some are carried by currents to the depth, deposited on the bottom and forming wintering algae mats. After the ice break, in spring, these mats begin to grow, and some of them are brought ashore, where they can become food for herbivorous fauna [114, 115]. In addition to wintering mats, after the ice melts, the first ephemeral species of algae develop at the water's edge, where they are later replaced by the next generation of algae. *Cladophora sp*. and *Ulva sp*. form the largest aggregations in the Gulf of Finland [116, 117].

Ice conditions in the eastern part of the Gulf of Finland are extremely dynamic. In very cold winters, it can be completely covered with ice up to its western border; in the warmest winters, ice is either absent or appears only in the Neva Bay and Vyborg Bay. The main spring stopovers of swans are observed in the southern part of the Gulf of Finland—near Kurgalsky Peninsula and large islands to the north (Moschny, Maly and Seskar). The second place of large migratory stops is located off the northern coast of the Neva Bay.

### Fecal sampling

Samples were collected in 2014–2019 during the swan migration counts, which were carried out along the southern coast of the Gulf of Finland and along the northern coast of the Neva Bay (once every five days on average in 2014–2017, once a week on average in 2018–2019). On the Kurgalsky Peninsula, material was collected along the coast (Fig. 1) from the point with coordinates 59.651138N, 28.024824E on the western shore to the point with coordinates 59.683055N, 28.228864E on the eastern shore. On the northern coast of the Neva Bay the material was collected in the area between the coordinates 59.997672N, 30.058179E and 59.992835N, 30.094696E. In the Lebyazhiy reserve samples were taken on the coast near the village of Chernaya Lakhta at the coordinates 59.981623N, 29.254141E. The samples were taken after determining the species of swans and long observation of birds standing on land or in shallow water up to 15–20 cm deep. Fecal samples were collected only in cases of precise species identification. This usually occurred in a plot no larger than 20 by 20 m after observing a monospecific group of swans resting on it for at least 1 h. To avoid repeated sampling from the same individual, only one sample per species was taken from one site at each visit. The distance between adjacent collection points was at least 50 m (in the vast majority of situations more than 100 m).

All samples were fixed with 5% formaldehyde solution and further examined under a binocular microscope. We used an identification guide of plant residues in peat [118] to determine the percentage of residues of a particular plant species. Each plant species has a distinctive pattern, color, and shape of cells in the undecomposed residues. A detailed description of the analysis is given in [106]. Each sample was examined twice, by an algologist and a vascular plant specialist. The quantitative values of the two specialists were reconciled after the sample processing.

### Statistical analysis

Since bird diets are affected by changes of food supply during a particular period of the annual cycle, we divided our data in two nearly equal time periods to minimize the influence of collection date on the results and to ensure that sample dates for different swan species were approximately consistent. In the first period, from February 20 to March 20, only two swan species, Mute Swan and Whooper Swan, were observed. In the second one, from 21 March to 28 April, all three species were recorded. All further analyses were performed for each period separately.

Sixteen samples of the Mute swan and the same number of samples of the Whooper swan for the first period (February 20—March 20), and 30 samples of each of the three swan species for the second period (March 21—April 28) were used in the study (Table 1). In fact, we collected significantly more fecal samples of the Bewick’s swan and Whooper swan (77 and 67, respectively), but since the diversity indices we use in niche size analysis, as well as species richness are all influenced by the sampling effort, we had to use only a portion of these samples so that the total number for different species was the same. The question of which of them to keep and which to remove was solved as follows: we selected samples with similar (close) dates of collection for different species.

**Table 1:** The number of samples collected in different years and time periods within a year

Time periods	2014	2015	2016	2017	2018	2019	Total
**Mute Swan**							
20 February – 20 March	2	8	6	0	0	0	16
21 March – 28 April	12	12	6	0	0	0	30
Total	14	20	12	0	0	0	46
**Bewick’s Swan**							
20 February – 20 March	0	0	0	0	0	0	0
21 March – 28 April	0	12	3	3	0	12	30
Total	0	12	3	3	0	12	30
**Whooper Swan**							
20 February – 20 March	2	10	4	0	0	0	16
21 March – 28 April	0	8	7	1	5	9	30
Total	2	18	11	1	5	9	46

Nutritional data were presented as mean percentage of species volume in samples [119–121]. Similarities and differences in dietary preferences were analyzed by Nonmetric multidimensional scaling (NMDS) using Bray–Curtis dissimilarity in R Statistics software version 4.2.2 [122], Vegan package [123]. Volumes (%) of food items in each sample were used as input values for the analysis. Data from different observation sites and years were combined.

Then, we applied MANOVA to 4 food categories found in swan samples: 1) algae, 2) roots, rhizomes and tubers of vascular submerged and coastal plants, *e.g*. *Phragmites australis*, *Bolboschoenus maritimus*, *Agrostis sp.* and others, 3) vegetative parts of vascular submerged and coastal plants, and 4) detritus. Arcsine square root transformation was applied to the proportions since the error distributions did not match those of the normal distribution [124]. Wilks’ lambda was used as the test statistic for the identification of essential effects. The results were considered significant at *P* < 0.05. Where multivariate tests were significant, a posteriori analysis was performed using Tukey tests to determine exactly which species were different from the others.

Additionally, we divided all food items according to their roughness into the following categories (in ascending order of roughness from 1 to 5): 1) macroalgae; 2) thin root papillae (of all plants), as well as vegetative parts of vascular submerged vegetation (*Zannichellia palustris*, *Stuckenia pectinata, Potamogeton sp., Myriophyllum heterophyllum*, *Zostera marina, Ruppia maritima*); 3) vegetative parts of semi-aquatic grasses (*Agrostis sp.*, *Calamagrostis*, *Phalaris arundinacea*, *Carex sp., Alisma plantago-aquatica* etc.) and *Stuckenia pectinata* tubers; 4) young shoots of semi-submerged vegetation (*Phragmites australis*, *Bolboschoenus maritimus, Schoenoplectus tabernaemontani*) and rhizomes of *Nuphar lutea*; 5) *Phragmites australis* rhizomes. We then calculated mean food roughness for each sample, weighted by the volume of species in the sample, and compared food roughness in swans' diets using a Kruskal–Wallis non-parametric test. Where the results were significant, a post-hoc analysis was performed to determine which levels of variable differed from each other (we used Dunn's test with the FSA package in R [122, 125].

The dietary niche width was estimated using Levins’ index [40, 121, 126]: *B* = *1 / Σp*_*i*_^*2*^, where *p*_*i*_ is the proportion of food item *i*. Hurlbert’s formula was applied to standardize the Levins index: *B*_*a*_ = *(B – 1)/(N – 1)*, where N is the total number of food items. *B*_*a*_ values range from 0 (minimum dietary niche width) to 1 (maximum dietary niche width).

The overlap in diet among species was measured with the symmetric niche overlap coefficient (Pianka’s index [121, 127, 128]): *O*_*jk*_ = *(Σp*_*ij*_* p*_*ik*_*) (Σp*_*ij*_^*2*^* p*_*ik*_^*2*^*)*^*−1/2*^, ${p}_{ij}$ and ${p}_{ik}$ represent proportional values of food items consumption for species j and k. This indicator quantifies niche overlap among a set of discrete categories and ranges from 0 (no overlap) to 1 (complete overlap).

Using null models in EcoSim 1.00 [129] we tested whether the observed niche overlap differs from what would be expected if swan species used food resources independently of each other. The randomization algorithm RA3 was applied; the number of replications was 1000.

## Results

**Fig. 2:**
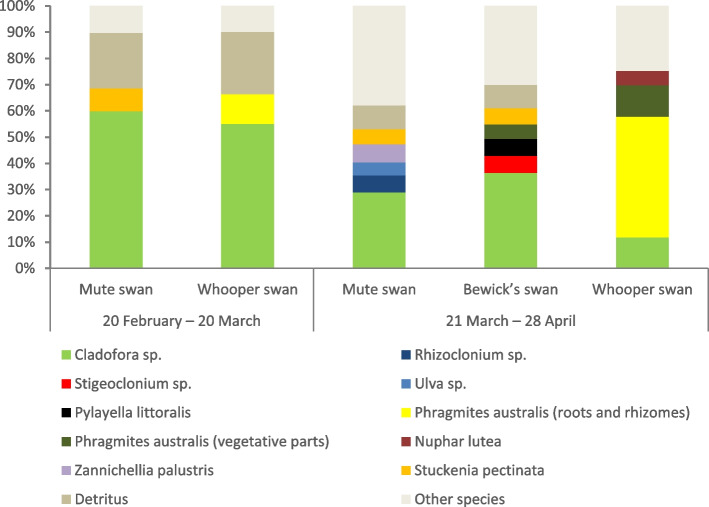
Main food items in the diet of swans (species with an average volume in samples greater than 5%)

Results of the analysis showed that between February 20 and March 20, Mute swans fed mainly on green algae (61%), predominantly *Cladophora sp*. (Table 2, Fig. 2, 60% of diet). Vegetative parts of pondweed *Stuckenia pectinata* (9%), thin root papillae of *Phragmites australis* (4%), and brown algae *Fucus sp.* and *Dictiota dichotoma* (3%) were present in much smaller amounts. The diet of Whooper swans during this period was very similar to that of the Mute swan: *Cladophora sp.* accounted for 55%, *Stuckenia pectinata* for 4%, and roots and rhizomes of reeds *Phragmites australis* for 11%. Detritus content in samples of both species was almost identical—21% (Mute swan) and 24% (Whooper swan). The other species were found in less than 3% (Table 2, Fig. 2). Fecal samples of Bewick’s swan were absent between 20 February and 20 March.

**Table 2:** Diet composition (volume % of species in the fecal samples) of Mute Swan, Bewick’s swan and Whooper swan in spring

Species	Mean % (s.e.)		
	**Mute swan**	**Bewick’s swan**	**Whooper swan**
**20 February – 20 March**			
**Chlorophyta**	**60.63 (4.03)**	-	**55.25 (6.38)**
*Chaetomorpha sp.*	0.13 (0.13)	-	0 (0)
*Cladophora sp.*	59.94 (4.09)	-	55.13 (6.43)
*Oedogonium sp.*	0.19 (0.19)	-	0 (0)
*Rhizoclonium sp.*	0.38 (0.26)	-	0.13 (0.13)
**Phaeophyta**	**2.63 (0.98)**	-	**1.81 (0.74)**
*Fucus sp.* + *Dictiota dichotoma*	2.63 (0.98)	-	1.81 (0.74)
**Bacillariophyta**	**0.69 (0.51)**	-	**0 (0)**
*Tabellaria fenestrata*	0.63 (0.51)	-	0 (0)
*Diatoma elongatum*	0.06 (0.06)	-	0 (0)
**Roots, rhizomes and tubers of vascular submerged and coastal plants**	**5.06 (1.62)**	-	**13.63 (5.33)**
*Phragmites australis*	3.75 (1.25)	-	11.25 (4.37)
*Bolboschoenus maritimus*	1.31 (0.55)	-	0.81 (0.45)
*Schoenoplectus tabernaemontani*	0 (0)	-	1.19 (0.79)
*Agrostis sp.*	0 (0)	-	0.38 (0.26)
**Vegetative parts of vascular submerged and coastal plants**	**9.88 (1.93)**	-	**5.56 (1.55)**
*Stuckenia pectinata*	8.69 (1.91)	-	4.06 (1.29)
*Potamogeton perfoliatus*	1.19 (0.42)	-	1.5 (0.96)
**Detritus**	**21.13 (4.4)**	-	**23.75 (6.51)**
**21 March – 28 April**			
**Chlorophyta**	**47.33 (4.85)**	**47.80 (6.98)**	**14.33 (5.53)**
*Chaetomorpha* sp.	2.3 (0.58)	0.1 (0.07)	0.07 (0.07)
*Cladofora* sp.	29 (5.54)	36.4 (5.83)	11.83 (4.86)
*Microspora amoena*	0.23 (0.2)	0 (0)	0.03 (0.03)
*Mougeotia* sp.	0.37 (0.19)	0.3 (0.19)	0.78 (0.36)
*Oedogonium* sp.	1.57 (0.48)	0.1 (0.07)	0.07 (0.05)
*Rhizoclonium* sp.	6.4 (3.49)	2.93 (1)	0.43 (0.24)
*Spirogyra* sp.	0.1 (0.07)	1.27 (0.62)	0.92 (0.39)
*Stigeoclonium* sp.	2.17 (0.76)	6.5 (3.83)	0.03 (0.03)
*Ulothrix zonata*	0.17 (0.08)	0.03 (0.03)	0 (0)
*Ulva* sp.	5.03 (1.88)	0.17 (0.17)	0.17 (0.17)
**Phaeophyta**	**0 (0)**	**6.43 (2.42)**	**0.03 (0.03)**
*Pylayella littoralis*	0 (0)	6.43 (2.42)	0.03 (0.03)
**Rhodophyta**	**4.3 (1.18)**	**0 (0)**	**0.07 (0.07)**
*Ceramium tenuicorne*	4.3 (1.18)	0 (0)	0.07 (0.07)
**Xanthophyta**	**0.9 (0.44)**	**0 (0)**	**0 (0)**
*Vaucheria* sp.	0.9 (0.44)	0 (0)	0 (0)
**Bacillariophyta**	**18.27 (2.33)**	**0 (0)**	**0 (0)**
*Melosira varians*	3.9 (1)	0 (0)	0 (0)
*Tabellaria fenestrata*	2.37 (0.39)	0 (0)	0 (0)
*Diatoma elongatum*	3 (0.48)	0 (0)	0 (0)
*Navicula* sp.	2.87 (0.44)	0 (0)	0 (0)
*Gomphonema constrictum*	2.47 (0.46)	0 (0)	0 (0)
*Cocconeis placentula*	2.93 (0.56)	0 (0)	0 (0)
Other Diatom species	0.73 (0.21)	0 (0)	0 (0)
**Roots, rhizomes and tubers of vascular submerged and coastal plants**	**6.7 (3.15)**	**11.20 (3.71)**	**52.83 (6.3)**
*Phragmites australis*	4.63 (2.01)	4.43 (1.15)	45.97 (6.51)
*Bolboschoenus maritimus*	2.07 (1.19)	0.27 (0.19)	2.4 (1.85)
*Schoenoplectus tabernaemontani*	0 (0)	0.83 (0.42)	1.67 (0.69)
*Agrostis sp.*	0 (0)	0.57 (0.4)	0.53 (0.37)
*Calamagrostis sp.*	0 (0)	0 (0)	0.03 (0.03)
*Phalaris arundinacea*	0 (0)	1.33 (1.33)	0.33 (0.33)
*Carex sp.*	0 (0)	1 (0.69)	0.48 (0.25)
*Eleocharis palustris*	0 (0)	0 (0)	0.42 (0.34)
*Stuckenia pectinata*	0 (0)	2.77 (2.43)	1 (0.84)
**Vegetative parts of vascular submerged and coastal plants**	**13.5 (3.66)**	**25.73 (7.44)**	**32.73 (6.07)**
*Phragmites australis*	0 (0)	5.5 (3.49)	12 (3.88)
*Bolboschoenus maritimus*	0 (0)	0 (0)	1.33 (0.76)
*Schoenoplectus tabernaemontani*	0 (0)	2.33 (1.59)	0.17 (0.17)
*Agrostis sp.*	0.7 (0.35)	0 (0)	0 (0)
*Phalaris arundinacea*	0 (0)	0.17 (0.17)	0 (0)
*Carex sp.*	0 (0)	0.33 (0.23)	0 (0)
*Alisma plantago-aquatica*	0 (0)	0 (0)	0.07 (0.07)
*Eleocharis palustris*	0 (0)	0 (0)	0.17 (0.17)
*Butomus umbellatus*	0 (0)	1 (0.5)	0.23 (0.18)
*Sparganium sp.*	0 (0)	3.33 (1.75)	3.67 (1.64)
*Sagittaria sagittifolia*	0 (0)	0.17 (0.17)	4.33 (2.14)
*Nuphar lutea*	0 (0)	4.5 (2.19)	5.5 (2.25)
*Hydrocharis morsus-ranae*	0 (0)	0 (0)	0.27 (0.19)
*Zannichellia palustris*	6.93 (2.19)	0.23 (0.18)	0 (0)
*Myriophyllum heterophyllum*	0 (0)	0.5 (0.5)	0 (0)
*Stuckenia pectinata*	5.73 (1.7)	6.27 (2.31)	4.23 (1.61)
*Potamogeton perfoliatus*	0.13 (0.08)	1.40 (0.58)	0.77 (0.4)
**Detritus**	**9 (3.24)**	**8.83 (3.21)**	**0 (0)**

From March 21 to April 28, the abundance of green algae in the diet of Mute swan decreased to 47%. Along with *Cladophora* (29%), feces contained *Rhizoclonium* sp. (6%), *Ulva* sp. (5%) and other green algae species; diatoms also appeared in large amount (18%). Compared with the previous period, the share of vascular plants increased (20%), *Zannichellia palustris* made the greatest contribution (7%). The proportion of green algae in the diet of Whooper swans strongly decreased to only 14% against the vascular plants domination (86%). Whooper swans began to eat mainly reeds, both rhizomes and leaves (the total share of *Phragmites australis* was 58%). *Nuphar lutea* appeared in the diet (6%), and the proportion of *Stuckenia pectinata* remained unchanged (4%).

In late March–April, numerous Bewick’s swans were present at migration sites. Green algae constituted a considerable portion (48%) of the fecal samples of this species, i.e. as much or even slightly higher than that of the Mute swan. Roots, rhizomes, and tubers of vascular submerged and coastal plants constituted 11% of the samples, and green parts of aquatic and coastal vegetation—26%.

### Width and overlap of dietary niches

**Fig. 3:**
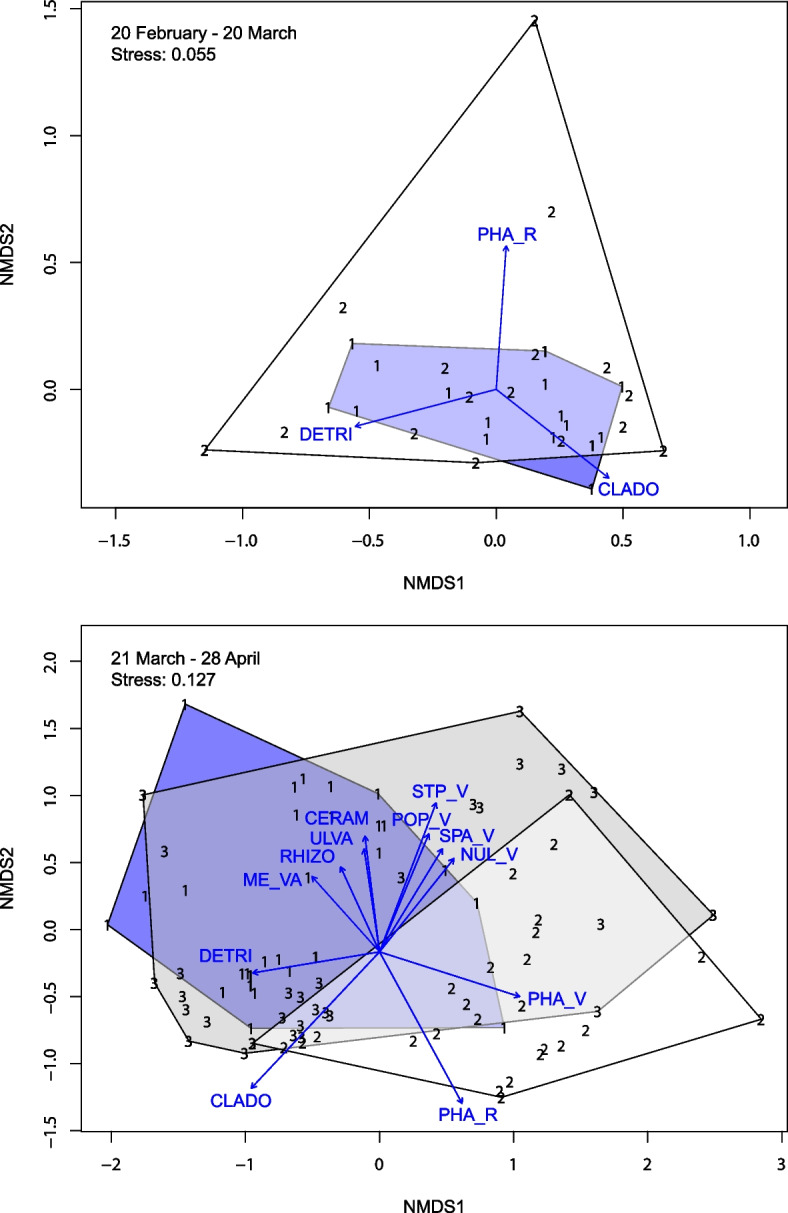
Non-metric multidimensional scaling (NMDS) using Bray–Curtis dissimilarity by the species composition for plant residues in fecal samples of swans. The numbers correspond to the species of swans: 1—the Mute swan (blue polygon), 2 – the Whooper swan (white polygon) and 3 – the Bewick’s swan (gray polygon). The arrows indicate the direction of the maximum contribution of the food items. The letter abbreviations refer to plant species and their parts: CLADO—Cladofora sp., PHA_R—Phragmites australis (roots and rhizomes), PHA_V—Phragmites australis (vegetative parts), SPA_V—Sparganium sp. (vegetative parts), STP_V—Stuckenia pectinata (vegetative parts), NUL_V – Nuphar lutea (vegetative parts), POP_V—Potamogeton perfoliatus (vegetative parts), ME_VA – Melosira varians, CERAM – Ceramium sp., ULVA – Ulva sp., DETRI – detritus

In February–March, numbers of food items and dietary niche width (Levins index) have similar values for the Mute swan and the Whooper swan (Table 3). The overlap of dietary niches between the Mute swan and the Whooper swan in the first period was 0.987 (P (Obs <  = null) = 0.001) (Table 4). Non-metric multidimensional scaling (NMDS) analysis also showed high overlap between diets of these two species (Fig. 3). However, Whooper swan’s cluster was broader and included the Mute swan. A similar pattern is shown by Levins index (the greater width of the Whooper swan niche), with the exception of the number of food items (the Mute swan has 2 more of them). Expansion of the Whooper swan diet was due to an increased consumption of the roots and rhizomes of *Phragmites australis*, as well as a little more detritus in samples (Fig. 3).

**Table 3:** Number of food items and dietary niche width for Mute Swan, Bewick’s Swan and Whooper Swan in 20 February – 20 March and 21 March – 28 April

	**Mute Swan**	**Bewick's Swan**	**Whooper Swan**
**20 February – 20 March**			
Number of food items	12	-	10
Levins’ index (standardized value)	2.42 (0.03)	-	2.66 (0.04)
**21 March – 28 April**			
Number of food items	26	30	32
Levins’ index (standardized value)	8.47 (0.16)	6.11 (0.11)	4.01 (0.06)

**Table 4:** Dietary niche overlap among the three swan species in 20 February – 20 March and 21 March – 28 April. *represents *P* < 0.05

Species	Observed niche overlap index	P (Obs < = null)	P (Obs > = null)
**20 February – 20 March**			
Mute Swan—Whooper Swan	0.987	0.001*	0.999
**21 March – 28 April**			
Mute Swan—Whooper Swan	0.344	0.969	0.031*
Mute Swan – Bewick’s Swan	0.883	0.999	0.001*
Whooper Swan – Bewick’s Swan	0.384	0.973	0.027*

The diet of swans naturally expanded from February to April due to the development of vegetation. In the second period (March 21-April 28) the niche width increased and was 8.47 in the Mute swan, 4.01 in the Whooper swan and 6.11 in the Bewick’s swan (Table 3). It is worth noting that there is a severe shortage of food resources in early spring. This is probably the reason why indicators are substantially lower in February–March than those in late March–April. The overlap decreased compared to the previous period (Table 4). The highest overlap was observed in Bewick's and Mute swans, 0.88 (P (Obs >  = null) = 0.001) (Fig. 3, Table 4). The lowest one was in the Mute and Whooper swan, 0.344 (P (Obs >  = null) = 0.031). Dissimilarity of diets of the latter two species in March–April was mainly due to transition of the Whooper swan to feeding on the reed *Phragmites australis* (both roots and rough rhizomes and vegetative parts of the plant, 58% of the diet in total, Table 2, Figs. 2 and 3). Mute swan, in turn, began to consume even more algae (their share increased from 64% in February–March to 71% in March–April), partially switching from green Chlorophyta to red *Ceramium* sp. (4.3% of diet) and diatoms (*Melosira varians, Tabellaria fenestrata, Diatoma elongatum, Navicula sp., Gomphonema constrictum* and *Sossonais placentula*—each comprising 2 to 4% of diet).

The diet of Bewick’s swans was similar in many respects to that of the Mute swan (Fig. 3), but Bewick’s swans much more often preferred vegetative parts of submerged plants, such as *Stuckenia pectinata, Potamogeton perfoliatus, Sparganium sp., Nuphar lutea*, as well as leaves of reeds *Phragmites australis* (Fig. 3, Table 2).

The difference in niche widths of the three swan species in March–April, calculated using the Levins index, in our opinion, is mainly due to the dominance of certain food categories.

### MANOVA

Additionally, we performed a multivariate analysis of variance (MANOVA) for 4 food categories: (1) algae; (2) roots, rhizomes, and tubers; (3) vegetative parts of vascular plants; and (4) detritus. For the first period (February 20—March 20), differences in food categories for the two species (Mute and Whooper swan) were not significant (MANOVA, Wilks' λ = 0.81, F(4, 27) = 1.53, *P* = 0.22), which is not surprising since there is very little available food in early spring. Apparently, swans have no choice, so they just eat whatever they come across.

**Fig. 4:**
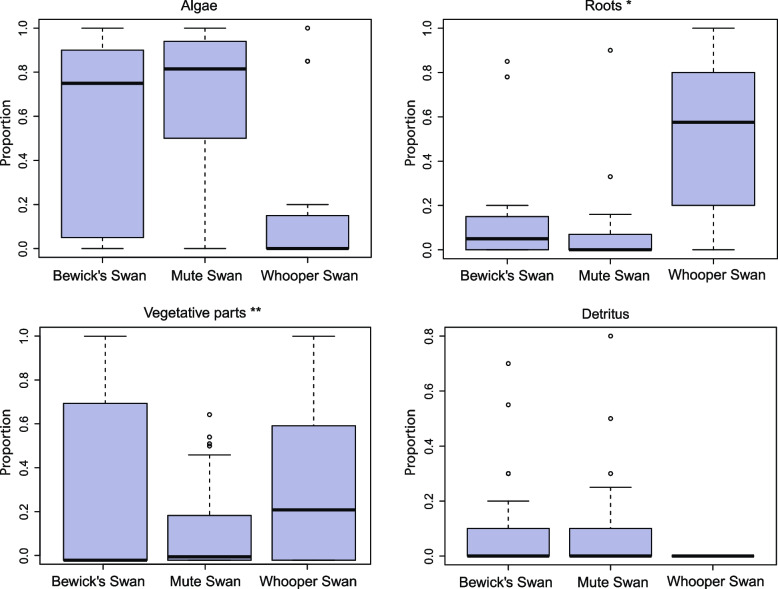
Boxplots of the four categories of food consumed by swans in 21 March—28 April. Horizontal line denote the median value, the whiskers represent minimum and maximum content of the food category in the sample, and the box is drawn from the first to third quartile. *Roots, rhizomes and tubers of vascular submerged and coastal plants. **Vegetative parts of vascular submerged and coastal plants

In the later period (March 20—April 28), the food content of the swan diets differed significantly (MANOVA, Wilks' λ = 0.49, F(8, 168) = 9.11, *p* < 0.001). A posteriori comparisons based on Tukey's tests showed no significant differences in the consumption of algae and roots and rhizomes by Mute and Bewick’s swan (*p* = 0.13), while the Whooper swan ate significantly less algae than the first two species (Fig. 4), and, instead, ate much more roots and rhizomes (*p* < 0.001 for both the Whooper swan—Mute swan pair and the Bewick’s swan—Whooper swan pair for these food categories) (Fig. 4). The content of vegetative parts of vascular plants in fecal samples of all three species did not differ significantly (*p* varied from 0.07 to 0.53 in all pairs). As for detritus, the lowest amount was found in samples of Whooper swan (*p* = 0.01 and 0.02 compared to Mute swan and Bewick's swan, respectively), but differences between Mute swan and Bewick's swan were not significant (*p* = 0.93).

It is interesting that detritus was almost absent from the Whooper swan samples in March–April, while in February–March it was a quite an essential part of the diet of this species (24%).

**Fig. 5:**
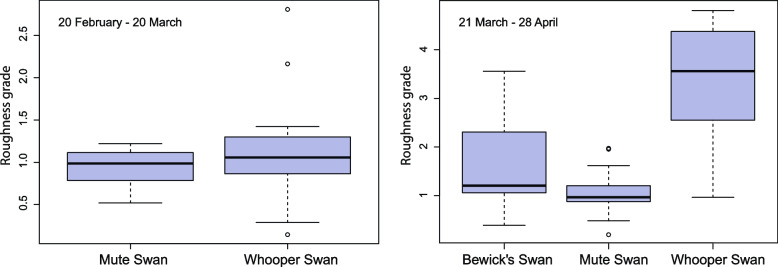
Comparison the diet of three swan species according to the roughness of food. Feed roughness grade varies from 1 to 5 (softest to roughest). Horizontal line denote median value, the whiskers represent minimum and maximum, and the box is drawn from the first to third quartile

Our last analysis focused on the roughness of the food consumed by swans. In the first period (February–March), the median roughness in samples of the Mute and Whooper swan did not differ significantly (${\chi }^{2}$ = 1.00, *p* = 0.32). In the second period (March–April) Kruskal–Wallis test was significant (${\chi }^{2}$ = 45.65, *p* < 0.001), so a post-hoc analysis was performed to determine which species diffed from each other. Dunn's test showed significant differences in all pairs of species. The roughness of the food in the diet of the Mute swan was expected to be the lowest (*p* = 0.02 for the Bewick’s swan—Mute swan pair and p < 0.001 for the Mute swan – Whooper swan pair), the highest in the Whooper swan samples, and the Bewick’s swan took an intermediate position (*p* < 0.001 for the Bewick’s swan – Mute swan pair) (Fig. 5).

## Discussion

The diet of swans in the eastern part of the Gulf of Finland differs in many ways from the diets of these birds in the more western parts of the Baltic and North Seas. First, we should note the almost complete absence in our results of *Zostéra marina* and *Ruppia maritima*, which are mass food for swans at sites in Sweden [32], since these plants are due to low salinity rarely found in the eastern part of the Gulf of Finland. Secondly, we did not notice any noticeable participation of tubers of *Stuckenia pectinata* in the diets of all species, which formed the basis in the late autumn and winter diets of swans wintering in the Netherlands [10, 14–17].

In our opinion, this is due to two factors. First, during wintering in the Netherlands, swans fed on tubers on coastal lakes and lagoons isolated from the sea and, accordingly, from the wave break, which contributed to the strong overgrowth of water bodies and a large abundance of this type of food. In the eastern part of the Gulf of Finland, all shallow waters are sufficiently open for wave activity, which does not contribute to strong pondweed overgrowth. In addition, in the most wave-protected areas, pondweeds often grow together with clumps of *Phragmites australis, Bolboschoenus maritimus* and *Schoenoplectus tabernaemontani*. These plants have very powerful developed rhizomes and form a dense sod cover, in which it is extremely difficult for birds to get tubers. Secondly, it is quite probable that in the spring, before the start of breeding, swans no longer need carbohydrate nutrition (tubers rich in starch), but protein, as happens, for example, in geese [36–39]. This force birds to consume young vegetative parts of plants.

Of course, tubers are difficult to detect in fecal samples, and the method we have adopted for recalculating the volume of tubers by the proportion of *Stuckenia pectinata* sprouts in samples does not exclude a certain underestimation of this food.

### Changes in swans' diets during the spring

The proportions of various foods vary greatly depending on the phenological period. Thus, in February–March, the main food of Whooper and Mute swans was *Cladophora sp.* which was carried by the currents to the shore. Scraps of pondweed, apparently, were eaten in small quantities by both species along the way from algal clumps. Probably *Cladophora sp*. becomes the main food not only because it is the most massive and easily accessible resource in the study area, but also because in late winter—early spring it is the only actively vegetative plant and is richest in amino acids, proteins, and other substances useful for birds.

In late March and April, when most groups of vascular submerged, semi-submerged and coastal vegetation begin to actively vegetate, Whooper swans and the Bewick’s swans begin to feed on these plants, eating their young shoots, which are the most nutrient-rich. *Phragmites australis* rhizomes dominate in the diet of Whooper swans, while Mute swans continue to feed mainly on algae. In the diet of the Mute swan, the share of Bacillariophyta strongly increases, in which the spring peak of abundance is observed at this time. Apparently, Bacillariophyta is eaten by swans along with multicellular filamentous algae on which they form dense growths. However, as we pointed out earlier [106], the content of a large amount of nutrients valuable for birds in diatoms is many times higher than in algae of other systematic groups [130]. That is, the appearance of diatoms in the diets of the Mute swan may play the same role as young shoots of higher vegetation in the diet of other species.

It should be noted here that in the diets of two other species of swans at that time, various filamentous algae were also present, but we did not find diatom remains in their faecal samples. The reasons for this are not completely clear and can be associated not so much with small sample sizes, but also with the fact that diatoms do not grow on all species of algae, the timing and intensity of their fouling also depend on the age of the substrate algae and local water temperatures in the area [131]. In addition, one cannot exclude the partial influence of the more advanced filtering apparatus of the beak of the Mute swan in comparison with other species of swans, and, consequently, the use of slightly different methods for absorbing food lumps [82, 83]. These questions can only be clarified through further research.

### Overlapping of dietary niches and its change during the spring

The feeding niche of Mute swan consists of the softest aquatic vegetation—macroalgae and vegetative parts of vascular submerged plants (*Zannichéllia palustris* and pondweeds). At the same time, more than 60% is made up of various algae species. It should be noted that the food niche of this species changes very little during the season. The diet of the other two species is more diverse, they are characterized by wide use of, besides algae and submerged vascular plants, also coarser semi-submerged and coastal higher vegetation (*Phragmites australis, Schoenoplectus tabernaemontani, Carex sp*. etc.). Young shoots of *Phragmites australis* play a significant role in the diet of both species. At the same time, the ration of whooper swans is most shifted towards the use of coarse fodder. Thick rhizomes of *Phragmites australis* were found only in the diet of Whooper swans, which constituted almost half of their diet in the end of March and April. The use of macroalgae in Whooper swans was about 5 times lower than in Mute swans and almost 4 times lower than in Bewick’s swans. Due to the aforementioned characteristics the food niche of Whooper swans is even more isolated from other species than that of Mute swans. It should be noted that the widespread use of reed by the whooper swan is a distinctive characteristic of its spring feeding in other areas as well [40].

As can be seen from our research, the trophic niches of the Mute swan and the Whooper swan were the narrowest and most overlapped in late winter—early spring, when food supplies were the poorest in species composition and quantity, compared with April, when most aquatic and coastal plant species began to grow. Later in April, the feeding niches of both species diverge significantly, primarily due to the transition of Whooper swans to feeding on vascular plants. Our results are in some contradiction with the data of Liu and co-authors [40], who claim that with a poorer food base, the species' food niches diverge to reduce inter-species competition [132–134]. Such a contradiction may be due to two circumstances. Firstly, it is not quite clear what the threshold value of the food shortage should be, at which there is a critical increase in interspecific competition that causes niche divergence. Secondly, at different stages of the annual cycle (wintering and preparation for breeding), the needs of birds for different feeds should change, which in itself can change the feeding niches of species and lead to their divergence.

### Differences in feeding niches as one of the factors determining the population dynamics of the three swan species

There is no doubt that such a strong preference for feeding on filamentous algae is one of the important factors that determined the expansion of the Mute Swan in the Baltic Sea region, as the development of the warm phase of climate and eutrophication of the Baltic Sea [135] provide this species with practically unlimited forage resource. At the same time, the ability to eat rougher food may give certain advantages to the Whooper Swan compared to the Bewick’s swan on wintering grounds and at migration stops, which may eventually determine the different direction of their population dynamics.

### The influence of morphological adaptations on the characteristics of the species' feeding niche

The differences in the food niches of the three species of Palearctic swans are in full agreement with their morphological adaptations. As mentioned above, the Mute swan, due to the minimum tarso-metatarsus length index, as well as the most pronounced filtrating features in the beak morphology, is the most aquatic form adapted to feeding from the bottom of soft submerged aquatic vegetation [2, 4, 81–83]. The Whooper and the Bewick’s swan, in which body proportions have some semi-terrestrial features, and in the morphology of the beak, “goose-like grazing” functions are more pronounced (ibid.), feed to a greater extent on coarse semi-submerged coastal and semi-submerged vegetation. At the same time, the coarsest food resource, *Phragmites australis* rhizomes, is developed exclusively by the Whooper swan, which has the most powerful beak, and the smaller Bewick’s swan, being the most “land form” with the smallest neck length index and the largest tarso-metatarsus length index, feeds more often than other swan species on land plants (families Poaceae, Cyperaceae, Butomaceae, etc.).

### Paleoecological prerequisites for the divergence of food niches and morphological adaptations of Palearctic swans

As can be seen from the analysis of paleontological findings, the formation of the Mute swan occurred mainly during the Miocene-Pliocene in the southern part of the Palearctic, presumably in the area covering the southern part of the East European Plain, the northern Black Sea region, the Caspian lowland and Central Asia [53, 54], possibly this region reached central Mongolia in the east [136]. The climate of these epochs was characterized as a gradual transition from the Miocene optimum to a much more arid and cooler at the end of the Pliocene, but in general, it was much warmer and more humid than at present [137–140].

In most of this territory, replacing each other, there were large sea basins, as parts of the Paratethys Ocean—the Pannonian, Sarmatian, Meotic, Akchagyl Sea, Lake Balakhani [141–145]. The salinity of these water bodies varied greatly up to complete desalination, depending on the opening and closing of the straits connecting them with the Indian Ocean and the Mediterranean Sea [142]. The coastline of these water bodies was strongly indented, there were numerous archipelagos of islands and lagoons [140–145]. Along the coasts of lagoons and islands, there were extensive, well-heated shallow water zones [140]. These epochs are characterized by a wide development of higher aquatic vegetation, both rigid semi-submerged (*Phragmites australis, Typha latifolia, T. angustifolia*) and soft submerged (*Potamogeton sp., Najas marina, Vallisneria sp., Ceratophyllum sp., Batrachium rionii, Myriophyllum sp., Ruppia maritima, R.spiralis, Zannichellia palustris,* and *Z. pedunculata*) and floating vegetation (*Salvinia sp., Lemna sp.,* and *Hydrocharis sp.*) [140, 146–148]. It should also be noted that due to the separation of Antarctica from South America, a cold circumantarctic current formed in the Miocene, which caused bottom waters rich in nutrients to rise to the surface of the world ocean, which, in turn, caused a massive increase in the number of algae in the shallow waters of all seas, including Paratethys, as well as the widespread occurrence of diatoms [130, 149, 150]. This whole complex of rich food conditions contributed to the formation of the Mute swan as a species specializing in feeding on aquatic submerged soft vegetation, which it consumes with the help of beak of a filtering type. An increase in body size and neck length contributed to the expansion of the zone with food available to the species, due to its deepest parts. The formation of species of Palearctic swans of the subgenus *Olor* (Whooper swan and Bewick’s swan) occurred during the Pleistocene with the greatest development of the temperature minimum and maximum aridization of the climate [151–153]. At that time, periglacial and thermokarst water bodies on permafrost, as well as dystrophic peat lakes among sphagnum bogs, became widespread in most of the Palearctic [139, 154]. Low water temperatures did not contribute to the strong development of higher submerged and algal vegetation in these water bodies (ibid.). However, along the banks of these water bodies, herbaceous coastal, marsh and semi-submerged vegetation develops and evolves—the families *Poáceae, Cyperaceae, Juncaceae, Alismatáceae, *etc*. *[148, 155–157].

Colonization of these habitats by young species of swans (subgenus Olor) determined a partial transition to feeding by the “goose type” (plucking out coarser grassy vegetation when grazing on the coast) and led to the appearance of some features of convergent similarity with geese—elongation of the tarso-metatarsus with some shortening of the neck and more powerful beak than that of a Mute swan with pronounced “grazing features” [2, 4, 81–83]. However, a necessary condition for their existence in the Pleistocene tundra steppes with a very short frost-free season is a significant reduction in the reproductive cycle and an increase in the growth rate of chicks, which requires the availability of feed with a high energy value and a high content of proteins and essential amino acids. The latter circumstance apparently determined the presence of a certain proportion of aquatic invertebrates in the diets of nestlings of these species [2–4, 6, 20].

The Bewick’s swan, which has settled in the northernmost habitats with the shortest summer and poor food conditions, consisting mainly of small grasses, sedges, rushes and horsetails, has the highest growth rate of chicks (about 2 months), the largest length index of the tarso-metatarsus with the smallest linear body dimensions and shortened neck [2, 4, 81]. The smaller overall size also determines the lower power of the jaw apparatus compared to the Whooper swan [82, 83], which is adapted to eating mainly small or soft coastal and aquatic vascular grasses.

Whooper swan lives in habitats with a longer frost-free period and higher temperatures, which determines the possibility of having a longer period of chick growth (about 3 months) and, accordingly, the possibility of having a larger body size and a more powerful jaw apparatus [2, 4, 81–83]. These habitats are characterized by the presence of more developed perennial semi-submerged and coastal vegetation with stiff stems and massive rhizomes containing a large amount of nutrients. The ability to feed on rhizomes is especially important for this species in the pre-nesting period when the vegetative parts of plants are just beginning to grow.

## Data Availability

The datasets analyzed during the current study are available in DSpace repository, http://hdl.handle.net/11701/43965.
