# Correction: Ecological and phylogenetic aspects of the spring diet of three palaearctic species of swans

**DOI:** 10.1186/s12862-024-02211-8

**Published:** 2024-02-14

**Authors:** Sergei A. Kouzov, Anna V. Kravchuk, Elena M. Koptseva, Yulia I. Gubelit, Elmira M. Zaynagutdinova, Evgeny V. Abakumov

**Affiliations:** 1 Department of Applied Ecology, St. Petersburg State University, Universitetskaya Emb. 7-9, St., Petersburg, 199034 Russia; 2 Department of Vertebrate Zoology, St. Petersburg State University, Universitetskaya Emb. 7-9, St., Petersburg, 199034 Russia; 3 Zoological Institute RAS, Universitetskaya Emb. 1, St., Petersburg, 199034 Russia

**Correction: BMC Ecol Evo 24, 17 (2024)**

10.1186/s12862-024-02204-7

In this article [1], the following statement was missing from the Funding section in the Declarations:

This research was funded by Saint Petersburg State University, project ID: 101,662,710 (CZ_MDF-2023-1).

The original article has been updated to reflect the correct statement.
